# A haploscope based binocular pupillometer system to quantify the dynamics of direct and consensual Pupillary Light Reflex

**DOI:** 10.1038/s41598-021-00434-z

**Published:** 2021-10-26

**Authors:** Najiya S. K. Meethal, Deepmala Mazumdar, Sergii Morshchavka, Jasper Robben, J. van der Steen, Ronnie George, Johan J. M. Pel

**Affiliations:** 1 Department of Neuroscience, Vestibular and Ocular Motor Research Group, Erasmus MC, Room EE 1453, PO Box 2040, 3000 CA Rotterdam, The Netherlands; 2 Medical and Vision Research Foundation, Chennai, India; 3 Zaporizhzhia Polytechnic National University, Zaporizhzhia, Ukraine; 4 Royal Dutch Visio, Huizen, The Netherlands

**Keywords:** Neuroscience, Health care, Medical research

## Abstract

This study described the development of a haploscope-based pupillometer for the parametrization of the Pupillary Light Reflex (PLR), and its feasibility in a set of 30 healthy subjects (light or dark-colored irides) and five patients diagnosed with Relative Afferent Pupillary Defect (RAPD). Our supplementary aim focused on evaluating the influence of iris colour on the PLR to decide whether a difference in PLR parameters should be anticipated when this system is used across ethnicities. All the participants underwent a customized pupillometry protocol and the generated pupil traces, captured by an eye tracker, were analyzed using exponential fits to derive PLR parameters. A Pupil Response Symmetry (PRS) coefficient was calculated to predict the presence of RAPD. The mean (SD) Initial PD during dilation (3.2 (0.5) mm) and the minimum PD during constriction (2.9 (0.4) mm) in the light iris group had a statistically significant (*p* < 0.001) higher magnitude compared to the dark iris group. The normal limits of the PRS coefficient ranged from − 0.20 to + 1.07 and all RAPD patients were outside the calculated normal limits. This proposed system, analysis strategies, and the tested metrics showed good short-term repeatability and the potential in detecting pupil abnormalities in neuro-ophthalmic diseases.

## Introduction

The photosensitive cells in the human retina are uniformly adapted to function over a wide range of ambient light conditions. When transitioning from various light intensity levels, Pupil Diameter (PD) undergo dynamic alterations elicited by Pupillary Light Reflex (PLR), thereby assisting in the process of visual adaptation. In addition, PLR also acts as a protective mechanism to safeguard the retinal cells from phototoxic damage caused by any exposure to intense visible light. In such instances, PLR results in a rapid change in PD, thereby optimising the influx of light that strikes the retina^1,2^. The PLR pathway is controlled by the integration of signals from the photoreceptors as well as the intrinsically photosensitive Retinal Ganglion Cells (ipRGCs), a subpopulation of Melanopsin containing RGCs that are sensitive to blue light. This reflexive process is directed by the antagonistic action of sphincter and dilator pupillae muscles subserved by the parasympathetic and sympathetic nerves, respectively. Hence, the analysis of PLR provides essential information regarding the integrity of these neural pathways^1–4^. Even though the key determinant of PLR is the level of retinal illuminance, additional factors such as age, gender, iris color, clarity of optical media of eye, the integrity of optic nerve, etc. are also found to have an effect ^5^.

Conventionally, the PLR is evaluated using a Swinging Flashlight Test (SFT), which is a qualitative assessment of the direct and consensual pupillary reflexes^1–3^. This deceptively simple approach is crucial and beneficial in routine clinical practice for defect detection and monitoring. However, it is prone to substantial inconsistency and inter-examiner variability due to its dependence on the examiner’s judgment and clinical expertise. Moreover, this method does not provide information regarding the delay, the speed, the extent, and the sustainability of pupil response. Thus, well-controlled systems are required to precisely quantify the parameters of both pupils^6–8^.

The technological advancements have led to multiple attempts to improve the objectivity of pupil response analysis by introducing pupillometer systems that aid in quantifying not just pupil diameters but also additional parameters that can be documented and monitored over time^9–21^. Currently, pupillometers are either commercially available and accessible pupillometers or laboratory-based prototypes. These are often designed for a specific clinical application or configured for a particular scientific study and differ from each other in their design, geometry, system characteristics, technical specifications, and range of applications. The proposed designs include monocular^9–13^ and binocular pupillometer systems^14–21^, that offer non-invasive, precise quantification of pupil parameters by relying on techniques such as CCD camera (Charge Coupled Device) detectors, firmware camera, video-based eye tracking technology, etc.

While intending to use such systems in a clinical or research arena, a binocular registration of pupil response is advised for various reasons. First, it is a diagnostically important information to quantify not only the direct PLR but also the consensual one. Next, the pupil responses are markedly variable over time; therefore, a simultaneous recording and comparison of inter-pupil activities are always reliable/preferred. Moreover, binocular assessment is considered to be a realistic imitation of a person’s life situations^21^. Though the literature has described various binocular pupillometers they are either inaccessible, expensive, inflexible, or less versatile to exclusively use for comprehensive pupil research^14–21^. Hence, we decided to customize a binocular pupillometer setup by integrating an infrared eye tracking device with a computer system for administering light stimulation. A previous study that evaluated pupillary reflex during perceptual rivalry used a binocular stimulus presented dichoptically on two monitors by projecting them with a mirror stereoscope to each eye separately. They could successfully quantify the pupil size and gaze direction using an infrared based eye tracking device placed behind the dissociating mirrors^22^. Yet another study described a detailed practical guide to build a specific eye tracker based set up integrated into a system with the possibility of an independent (dichoptic) visual stimuli presentation to each of the two eyes^23^. Relying on a similar concept, we developed a haploscopic system to control the laterality and the spatial extent of retinal stimulation and to further extend the possibility of the intended system to display three-dimensional simulation. The haploscope is an optical device that enables the dissociation of two eyes and the projection of two separate images to each of the observer's eyes^22–24^. It offers the option for simultaneous, unilateral, and alternating pupillary light stimulation (named as stimulation phases) whereas the real-time monitoring and recording of pupillary responses can be done using the eye tracking device. Most (commercial) pupillometers report parameters such as minimum and maximum PD and average contraction speed. Here, we analyzed the pupil response signals obtained during each phase using exponential fitting procedures to extend the list of parameters with time-related pupil parameters, such as latencies and time constants, during dilation, constriction, and even redilation (pupil recovery/escape).

The aim of this study was to describe (i) the design and development of a haploscope-based binocular pupillometer system, (ii) the description of a double exponential fit function in the parametrization of the PLR, (iii) and the first-line evaluation of the system’s feasibility and applicability in a set of healthy subjects and patients diagnosed with Relative Afferent Pupillary defect (RAPD). Following this, we present two sample applications of the system as a proof of concept: (i) quantification of pupil dynamics during redilation responses (ii) the establishment of an asymmetry limit to predict the presence of RAPD. Even though the previous literature has reported the effect of factors such as iris colour on pupil dynamics, the reported findings are contradictory^5,25–28^. Our supplementary aim focused on evaluating the effect of iris colour on PLR characteristics. This comparison was expected to help us in deciding whether a difference in pupil parameters should be anticipated when our customized system is used across ethnicities.

## Materials and methods

### Study participants

Study participants included a total of 30 healthy subjects and 5 patients diagnosed with RAPD. The group of healthy subjects constituted volunteers aged between 20 to 29 years, recruited at two institutes: 1) the Erasmus Medical Center, Rotterdam, The Netherlands, (source of subjects with light coloured irides, n = 15) and 2) at a tertiary eye care centre, Sankara Nethralaya, Chennai, India, (source of subjects with dark coloured irides, n = 15). This subject group was defined as those with Best Corrected Visual Acuity (BCVA) of 20/20 for distance and N6 for near, ≤  ± 3.00 DS and <  − 2.00 DCyl of spherical and cylindrical ametropia respectively, normal pupil morphology and reactivity with no RAPD, and a healthy anterior and posterior segment. A detailed clinical history was obtained to rule out any systemic conditions/surgery/trauma or intake of pharmacological elements that would have a potential effect on pupil reactivity. The latter group had 5 patients diagnosed with neuro-ophthalmic diseases and an associated RAPD, recruited from the neuro-ophthalmology Department, Sankara Nethralaya, Chennai. Written informed consent was obtained from all the participants before enrollment. The study adhered to the declaration of Helsinki for research involving human participants^29^ and the clinical procedures were reviewed and approved by the Medical Ethics Committee of Erasmus University Medical Centre, Rotterdam, The Netherlands, and the Institutional Review Board of Vision Research Foundation, Chennai, India.

After the preliminary clinical history, all the healthy subjects underwent pupillary evaluation using SFT by an experienced examiner performed to assess the direct and consensual PLR. A flashlight was shone onto the right eye followed by the left eye pupils from the inferotemporal quadrant from an approximate distance of 10 cm. Extent and rapidness of pupil constriction and dilation were evaluated and this was used as a screening process to rule out the presence of RAPD. Meanwhile, for the five neuro-ophthalmic patients, a comprehensive ophthalmic evaluation was performed (according to the routine clinical protocol), followed by SFT and quantification of the severity of RAPD using a Neutral Density Filter (NDF). Next, an external photograph of the iris was taken by a single examiner using a 12-megapixel camera equipped with the six-element lens under standardized illumination conditions and camera settings. If necessary, the eyelids were lifted to eliminate shadowing. A previously published iris colour classification system was used as a reference set to grade iris pigmentation^27^. The photographs were subjectively ranked into five categories from grade 1 to grade 5 (blue, blue-green, green–brown, light brown, and dark brown) independently by two observers with no colour deficiencies (assessed using Ishihara pseudo-isochromatic plates). The final iris colour grade was determined by calculating the median scores of these two observers.

### The haploscope-based pupillometer

**Figure 1:**
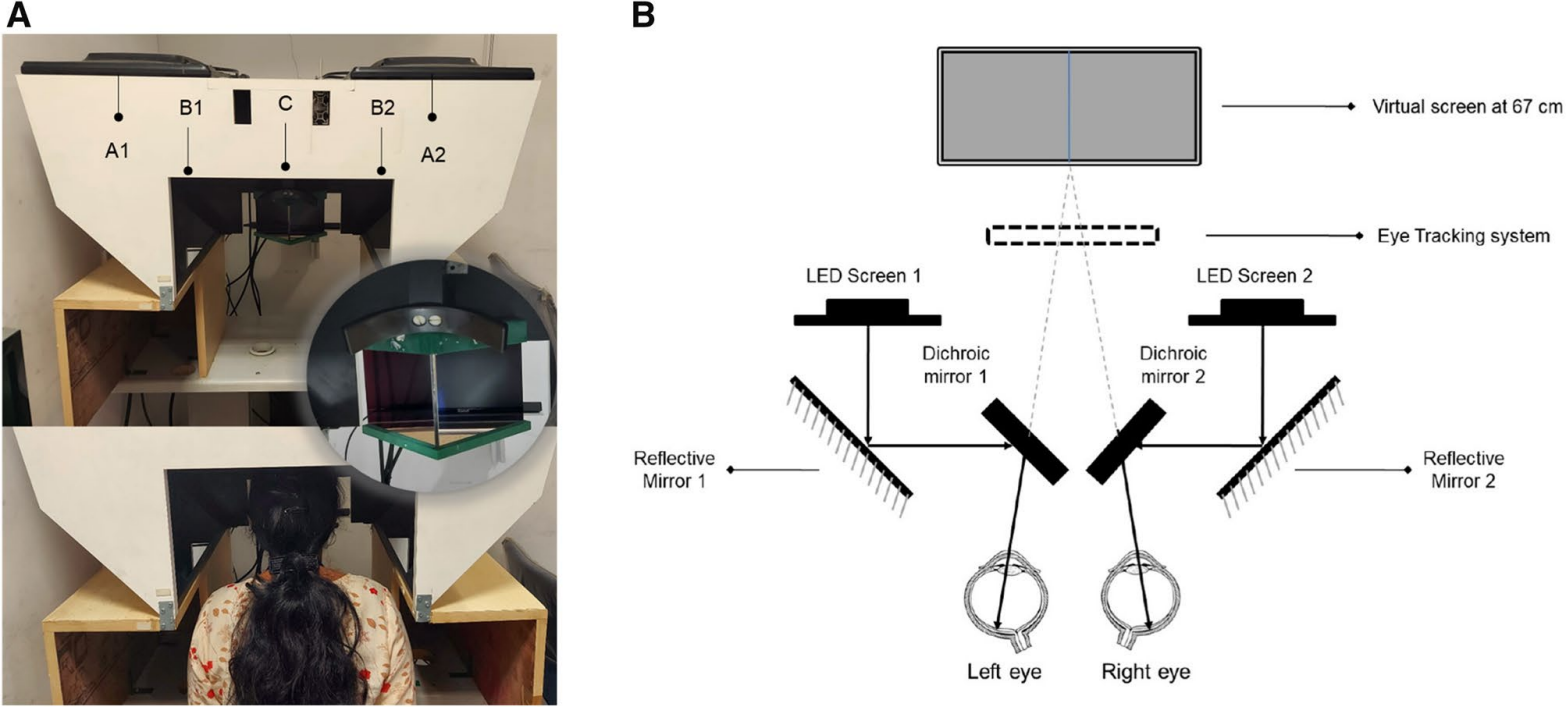
(**A**) Top panel: Image of the lab-based prototype of the binocular pupillometer. A1 and A2—LED screens 1 and 2, B1 and B2—the reflective mirrors, C—the cold mirrors inclined at an angle of 45 degrees. Middle panel: The zoomed in picture of the cold mirror and headrest. Bottom panel: Healthy subject with the head positioned at the device’s headrest. (**B**) The Schematic illustration of Haploscope-based pupillometer system that shows the image acquisition module with an infrared-based eye tracker and two Light Emitting Diode display monitors with two identical dichroic/cold mirrors in the optical path.

All the study participants underwent pupillometry using the customized test protocols incorporated in the haploscope. We had two identical setups with respect to their geometry, optical aspects, hardware, and software, one available in each lab (Fig. 1A). The system is comprised of an image acquisition module integrated with a customized test paradigm. The image capture unit consisted of an infrared-based eye tracker (Tobii Pro X3-120: sampling rate of 120 Hz for gaze and 40 Hz for pupil size) and two Light Emitting Diode (LED) displays for stimulus presentation. Two identical dichroic/cold mirrors (dimension: 101.0 mm × 127.0 mm) inclined at an angle of 45 degrees were placed in front of the right and the left eye, which had a dual purpose. Firstly, the multi-layered dielectric coating of these mirrors reflected 90% of the visible light while transmitting 80% of infrared radiations thereby ideally reducing the undesirable heat emitted by the IR based camera. Secondly, they acted as a beam splitter to dissociate the eyes to create a haploscopic stimulus presentation (Schematic illustration in Fig. 1B). The Psychtoolbox-3 package^30^ was used to create stimulation and to provide a mechanism for accurate timestamps and synchronous changes in stimuli on screens. Synchronization was carried out both between the right and left screens and with timestamps, provided using the Tobii Pro X3-120 device. An auxiliary PC unit with an inbuilt video card (NVIDIA—ION graphics processor) was used to control the test paradigm and store the acquired data. The high-definition video card supported a superior quality visual display, rapid graphics processing, and image rasterisation. The participant’s head was positioned at the device’s headrest where they had to remain in a stable viewing position and orientation with respect to the eye tracker. Each participant was instructed to rest his/her forehead on the mount and to look into the virtual screens through the two dichroic/cold mirrors.

### Customized pupillometry test paradigm

**Figure 2:**
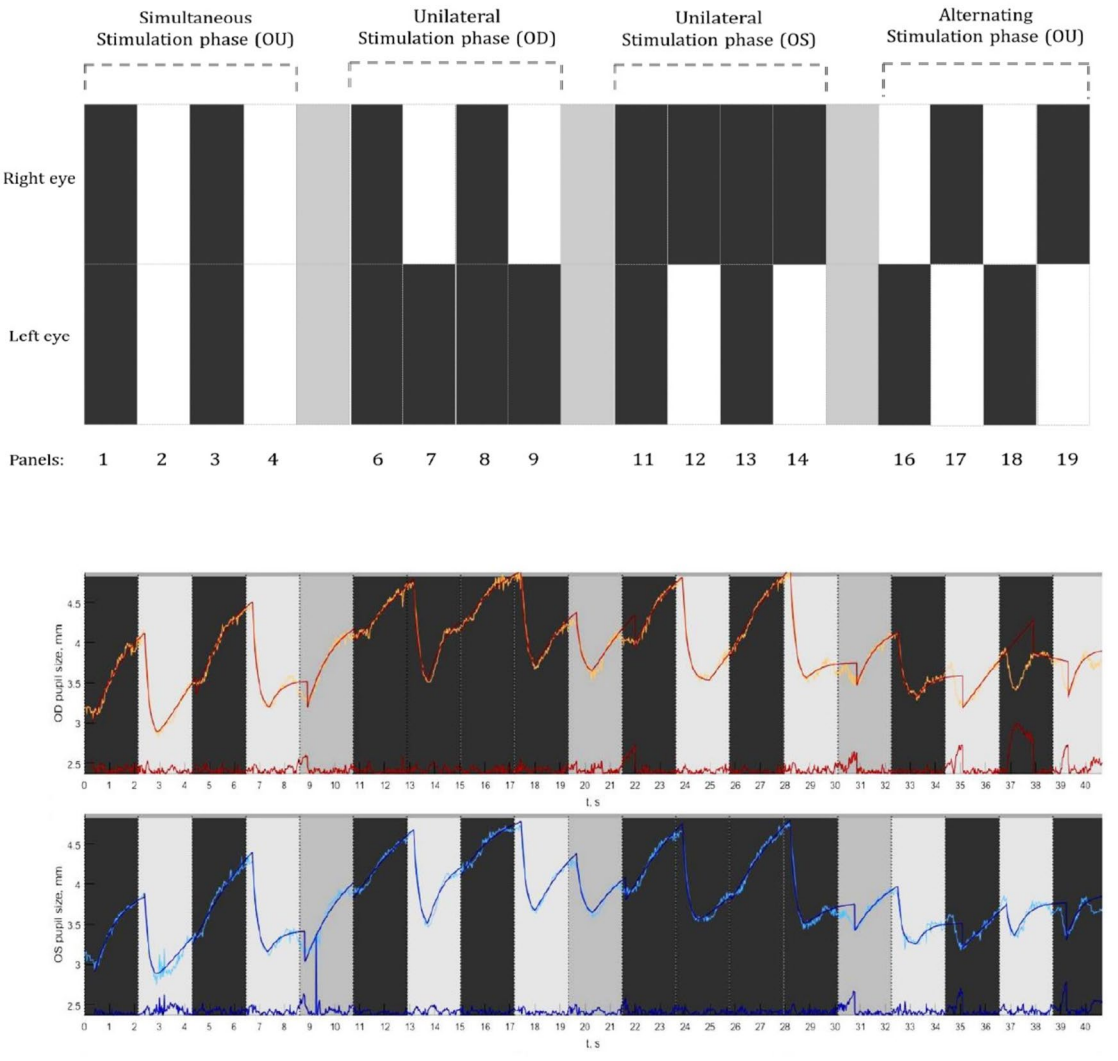
Top panel: A graphical illustration of the pupil stimulation protocol consisting of three stimulation phases. Simultaneous stimulation phase where both the eyes were stimulated; the unilateral stimulation phase where only one eye was stimulated followed by the other; and an alternating stimulation phase where both the eyes got alternatively stimulated. Bottom panel: Example of a pupillogram generated from the recordings of a healthy subject displaying the raw pupil traces from the right eye (in orange) and left eye (in blue). The superimposed traces are the best fit lines based on the double exponential fitting procedure along with the residual errors (red and blue traces displayed at the bottom).

The testing took place in a dark room and the light from the LED display was the only source of illumination. Each measurement began with an inbuilt five-point calibration procedure. After a successful calibration, each participant was instructed to look straight by fixating steadily at a Maltese cross target that was presented at the middle of an elliptical-shaped patch of light that appeared at the center of the screen. Next, a computerized pupil stimulation protocol was introduced (Fig. 2: Top panel). This customized protocol was divided into three sections, denoted as stimulation phases namely, (a) Simultaneous stimulation phase (Sim OU), (b) Unilateral stimulation phase (Uni OD and Uni OS), and (c) Alternating stimulation phase (Alt OD and Alt OS). During the first phase (Sim OU), both the eyes received light stimulation simultaneously, whereas, in the second phase (Uni OD and Uni OS), only one eye received light stimulation at a time. During the third stimulation phase, both the eyes received light stimulation in alternative turns. Light stimulation was made possible by projecting a white screen (RGB: 255:255:255), referred to as the white panel (illuminance at the eye level ranging from 109–115 Lux), and the removal of the light stimulation was made possible by projecting a black screen (RGB: 0–0–0), referred to as the black panel (illuminance at the eye level ranging from 0–2 Lux). So in brief, each of the stimulation phases consisted of four stimulation panels either white or black for light stimulation and withdrawal of light stimulation respectively. Each stimulation panel lasted for 2.7 s. In between each of the phases, a resting panel of 2.7 s (RGB: 169:169:169) was shown to minimize the effect of one cycle on the other. During the stimulation phases, participants were instructed to refrain from blinking, but during the resting panel (grey), they were allowed to blink if necessary. The entire stimulation protocol consisted of 19 stimulation panels that lasted for a total duration of 51 s. Subjects underwent two measurement series for PLR within an interval of ~ 5–10 min in which the initial one was considered as a baseline measurement to evaluate the inter-test repeatability.

### Data processing and response analysis

The data that was collected using the eye-tracker consisted of gaze positions and PD traces. The data on pupil dynamics were imported into a custom-made MATLAB program and processed to yield a pupillogram. It consisted of raw PLR response curves for each eye with PD plotted (y-axis) as a function of time in seconds (Fig. 2: Bottom panel). The raw traces were superimposed with the best fit lines obtained through the exponential fitting procedures (Fig. 2: Bottom panel). The pupillogram also displayed the amount of residual error of each exponential fit, by calculating the Root Mean Square Error (RMSE) values. This exponential fit function was used to analyze the time course of the pupil trajectory and the quantify PLR parameters from the double exponential equations (Appendix 1).

**Figure 3:**
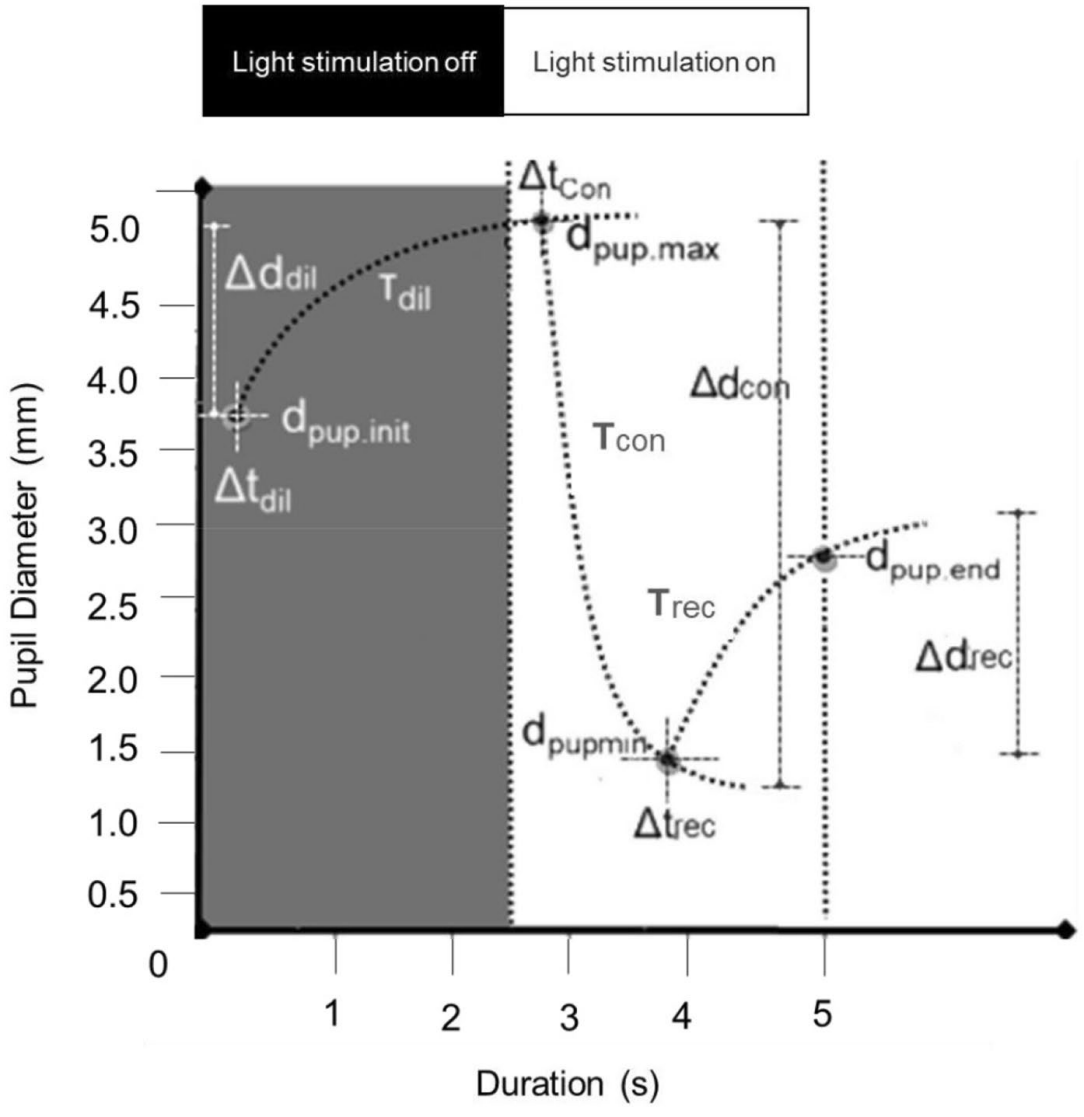
Shows the schematic diagram of a PLR curve and illustrates each of the measured parameters. PLR parameters derived were: d_pup.init_—The initial pupil diameter in a panel, d_pup.max_—The maximum pupil diameter in a dilation panel, d_pup.min (c)_—The minimum pupil diameter in a constriction panel, d_pup.end (c)_—The pupil diameter at the end of a redilation period in a constriction panel, Δd (amplitude i.e. absolute values of changes)—The difference between d_pup.max_ or d_pup.min_ and the d_pup.init._, Δt_dil_—The time delay/latency for the initiation of dilation after light stimulation is off, Δt_con_—The time delay/latency for the initiation of constriction after light stimulation is on, Δt_rec_—The time delay/latency for the initiation of pupil redilation after the maximum constriction. T_dil_, T_con_, T_rec_—The time constants (tau) representing the elapsed time required for the pupil to reach ~ 60% of its end value during dilation, constriction, and redilation.

From the Eq. 1 to 5 (Appendix 1) we calculated ‘d_pup_’ the PD varying from minimum (min) to maximum (max) diameter, ‘Δd’ the change in diameter during a stimulation phase (the direction of change was taken into account), ‘t’ the duration of a response, ‘Δt’ the latency for the PD change and ‘T’ the time constant (time for the pupil to reach ~ 60% of its end value) related to the speed of PD change (Fig. 3). Finally, the PRS coefficient (Appendix 1—Eq. 6) was calculated to characterize the inter-pupillary symmetry during constriction on the basis of constriction latency, speed, and amplitude. Here, the normative limits (mean ± SD) of PRS were calculated for each of the constriction panels from the group of healthy subjects and was compared with that of five patients with clinically diagnosed RAPD.

### Statistical analysis

Descriptive analysis of the demographic data was followed by the inspection of those parts of the obtained signal that resulted in a fit error > 0.2. A descriptive analysis of the error scores calculated for the exponentially fitted function revealed a definite cluster of 91.6% of the residual errors within 0.2 × 0.2 interval for the dilation and constriction panels ensuring an optimal quality of the data. Kappa-statistics was used for assessing the inter-observer agreement for iris colour grading. Participants with iris colour grade 1 to 3 were labelled as ‘light iris group’ whereas grades 4 and 5 were labelled as ‘dark iris group’.

Pearson's correlation was done for the inter-ocular comparison of the PLR parameters during dilation and for constriction. Intraclass Correlation Coefficient (ICC) was used as an index for repeatability that reflected both the degree of correlation and agreement between baseline and repeated measurements. An independent t-test was carried out to compare the diameter-related and time-related pupil dilation and constriction parameters between light and dark iris groups. One-way ANOVA was carried out to compare the diameter and time-related pupil constriction parameters to evaluate the constriction and redilation characteristics during bilateral and unilateral light stimulation and the post-hoc evaluation was carried out by applying the conservative *p*-value. An Independent t-test was carried out to compare the temporal characteristics of redilation with dilation responses. For each healthy subject, the PRS coefficient was calculated for OD and OS based on Eq. 6 (Appendix 1) and the inter-pupillary difference was obtained (OD–OS) from which the mean PRS and SD were calculated. The inter-pupillary difference in PRS coefficient was calculated for five RAPD patients and descriptively compared with the expected normal limits.

## Results

A total of 29 healthy participants with a mean age of 24 years (SD: 3) were enrolled which consisted of 21 females (72%) and 8 males (28%). The iris colour group classification comprised of 15 (52%) light iris (blue-green and green–brown) and 14 (48%) dark iris participants (Light brown and dark brown). One participant from the dark iris group was excluded due to a pupillogram with a residual fit error > 0.2. The inter-observer agreement for iris colour grading was excellent with a kappa coefficient of 1.0.

### PLR parameters during dilation response as a function of Iris colour and inter-ocular comparison

Two panels in the simultaneous stimulation phase i.e. Panel 1 and 3 (bilateral stimulation with black panels) were considered for comparing the PLR parameters during dilation. Table 1 summarizes the five different dilation parameters for the right and left eye and their statistical comparison between light and dark iris groups. A strong positive correlation (r = 0.7 to 0.9, *p* < 0.05, Pearson’s correlation) of all the dilation parameters between the right eye and left eye suggested no inter-ocular asymmetry. ICC coefficient values ranged from 0.81 to 0.92 for the dilation parameters, which showed a good agreement between baseline and repeated measurements with respect to dilation responses. For panel 3, a significantly (*p* < 0.01) higher magnitude of Initial PD (d_pup.ini_ (dil)) was found in the light iris group when compared to the dark iris one. Other dilation parameters including Time constant and latency were closely comparable irrespective of the stimulation panels (Panel 1 and 3).

**Table 1:** Displays the statistical comparison of dilation parameters obtained from stimulation panel 1 and panel 3 between light and dark iris participants.

Mean dilation parameters (SD)		Right eye			Left eye		
		Light Iris (n = 15)	Dark Iris (n = 14)	*p*-value	Light Iris (n = 15)	Dark Iris (n = 14)	*p*-value
Panel 1	d_pup.init_ (mm)	3.5 (0.7)	3.2 (0.6)	0.25	3.4 (0.6)	3.3 (0.5)	0.57
	d_pup.max_ (mm)	4.7 (0.8)	4.3 (0.4)	0.07	4.6 (0.7)	4.3 (0.4)	0.11
	Δd (mm)	1.2 (0.3)	1.2 (0.3)	0.57	1.2 (0.3)	1.1 (0.2)	0.65
	Δt (ms)	310 (170)	290 (160)	0.41	320 (165)	280 (160)	0.30
	T(ms)	1420 (495)	1285 (400)	0.60	1425 (420)	1200 (450)	0.80
Panel 3	d_pup.init_ (mm)	3.2 (0.5)	2.7 (0.3)	< 0.01	3.2 (0.5)	2.7 (0.4)	< 0.01
	d_pup.max_ (mm)	4.5 (0.7)	4.2 (0.3)	0.17	4.5 (0.8)	4.2 (0.4)	0.21
	Δd (mm)	1.3 (0.3)	1.5 (0.3)	0.08	1.4 (0.4)	1.6 (0.3)	0.15
	Δt (ms)	285 (130)	310 (110)	0.67	275 (140)	310 (100)	0.69
	T(ms)	1600 (445)	1260 (450)	0.08	1580 (375)	1320 (440)	0.14

Diameter-related pupil parameters expressed in millimeters (mm).

Time-related pupil parameters expressed in milliseconds (ms).

*SD* Standard Deviation, *p*-values are obtained from the Independent *t*-test.

### PLR parameters during constriction response as a function of Iris colour and inter-ocular comparison

Panel 2 and 4 (bilateral stimulation with white panels) were used to compare the PLR parameters during constriction. Table 2 summarizes the six different constriction parameters for the right and left eye and their statistical comparison between light and dark iris groups. A strong positive correlation (r = 0.7 to 0.9, *p* < 0.05, Pearson’s correlation) of all the dilation parameters between the right eye and left eye suggested no inter-ocular asymmetry. The calculated ICC coefficient values were found to be ranging from 0.86 to 0.96 for the constriction parameters, which showed a good agreement between baseline and repeated measurements with respect to constriction responses. For both the panels, a significantly (*p* < 0.01) higher magnitude of minimum PD (d_pup.min_ (con)) was found in the light iris group when compared to the dark iris group. Other constriction parameters including latency, duration, and time constant were statistically comparable irrespective of the stimulation panels (Panel 2 and 4).

**Table 2:** Displays the statistical comparison of constriction parameters obtained from stimulation panel 2 and panel 4 between light and dark iris participants.

Mean constriction parameters (SD)		Right eye			Left eye		
		Light Iris (n = 15)	Dark Iris (n = 14)	*p*-value	Light Iris (n = 15)	Dark Iris (n = 14)	*p*-value
Panel 2	d_pup.init_ (mm)	4.7 (0.7)	4.3 (0.4)	0.07	4.8 (0.8)	4.3 (0.4)	0.12
	d_pup.min_ (mm)	2.7 (0.4)	2.4 (0.3)	< 0.01	2.8 (0.4)	2.4 (0.2)	< 0.01
	Δd (mm)	1.9 (0.4)	1.8 (0.3)	0.60	1.8 (0.3)	1.9 (0.4)	0.46
	t (ms)	949 (274)	971 (237)	0.25	934 (239)	966 (271)	0.10
	Δt(ms)	255 (30)	260 (30)	0.47	250 (50)	240 (40)	0.68
	T(ms)	250 (40)	260 (50)	0.67	250 (50)	260 (60)	0.23
Panel 4	d_pup.init_ (mm)	4.7 (0.8)	4.3 (0.3)	0.08	4.7(0.9)	4.3 (0.4)	0.10
	d_pup.min_ (mm)	2.9 (0.4)	2.3 (0.3)	< 0.01	2.7 (0.4)	2.3 (0.3)	< 0.01
	Δd (mm)	1.9 (0.4)	2.1 (0.3)	0.27	1.9 (0.5)	2.0 (0.4)	0.61
	t (ms)	795 (197)	784 (203)	0.39	788 (252)	814 (258)	0.34
	Δt (ms)	225 (40)	250 (30)	0.16	235 (50)	250 (35)	0.62
	T (ms)	250 (40)	260 (35)	0.67	245 (45)	260 (35)	0.21

Diameter-related pupil parameters expressed in millimeters (mm).

Time-related pupil parameters expressed in milliseconds (ms).

*SD* Standard deviation, *p*-values are obtained from the Independent t-test.

### Temporal characteristics of pupil dynamics: comparison between dilation and constriction responses

Two dilation and two constriction panels in the simultaneous stimulation phase were used for comparing the time-related pupil parameters during dilation and constriction.

#### Time constant values

**Figure 4:**
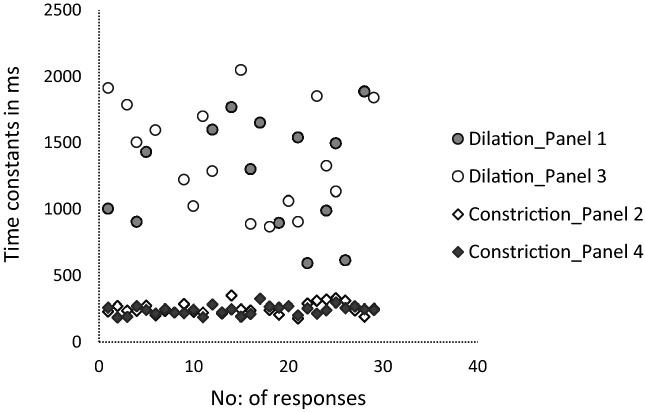
The scatter plot generated using number of partcipants/responses and Time constant values in the X and Y-axes respectively displaying higher Time constants for dilation in comparison with the constriction panels.

The mean time constant value for dilation was 1260 (SD: 420) ms whereas it was 250 (SD: 50) ms for constriction. Figure 4 illustrates an evidently higher Time constant values for dilation responses when compared to that of constriction which was statistically significant (One-way ANOVA, *p* < 0.001). The post hoc (Bonferroni) tests showed a significant difference between the two dilation panels in comparison with the two constriction panels.

#### Latency values

**Figure 5:**
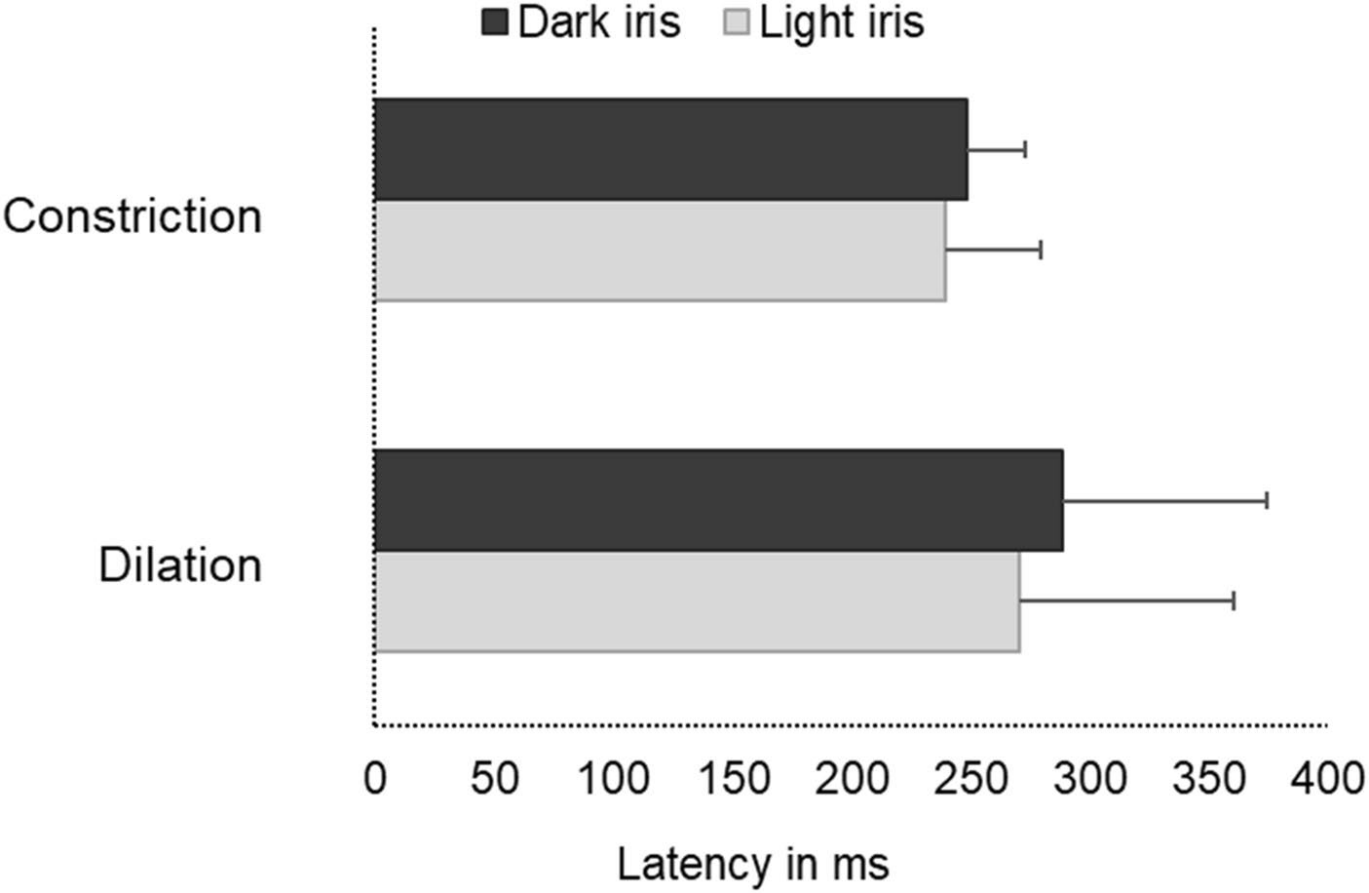
A cluster bar graph illustrating the delayed latency (milliseconds) during dilation in comparison with that of constriction among dark and light iris groups. Error bars represent the Standard Deviation (SD).

Figure 5 illustrates markedly delayed latencies during the dilation when compared to the constriction responses which was statistically significant (Mean difference: 35 ms, Independent T-test, *p* = 0.01) irrespective of their iris colour.

### Constriction characteristics: comparison between bilateral and unilateral photic stimulation

This analysis focused on constriction panels in the simultaneous stimulation phase (Sim OU) and unilateral stimulation phase (Uni OD and Uni OS).

#### Diameter-related parameters

The comparison of minimum PD (d_pup.min_ (con)) between the stimulation panels was found to be statistically significantly higher (Mean difference: − 0.3 mm, Independent t-test, *p*-value: 0.02) during simultaneous stimulation phase in contrast to the unilateral stimulation phase. Rest of the diameter-related constriction parameters such as initial PD (d_pup.init_ (con)) and PD amplitude (Δd (con)) were statistically not significantly different.

#### Time-related parameters

No statistically significant difference was found between the six panels (Supplementary Fig. 1) with respect to the Time constants (One-way ANOVA *p* = 0.41) as well as the latencies (One-way ANOVA *p* = 0.62).

### Redilation characteristics: comparison with dilation and between bilateral and unilateral photic stimulation

**Figure 6:**
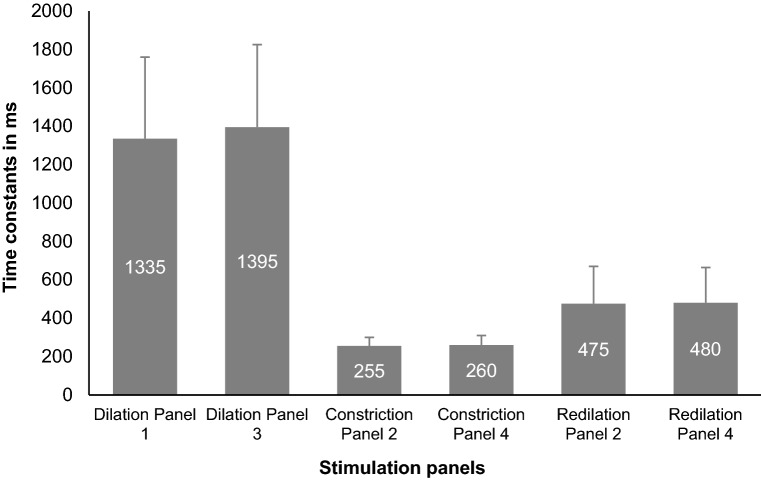
Bar graphs displaying the comparison of the Time constant values of constriction, dilation and redilation between the six stimulation panels. The error bars represent the Standard Deviation (SD).

A statistically significant difference (Independent *t*- test, *p*-value: < 0.001) in Time constant values was found between redilation (mean ± SD: 620 ± 320 ms) and dilation responses (mean ± SD: 1360 ± 455 ms), revealing a faster redilation compared to the usual dilation process (Fig. 6). The mean Time constants between the six panels ranged from 630 to 675 ms (mean ± SD: 650 ± 335 ms) where there was no statistically significant difference between the panels (One-way ANOVA *p* = 0.73), hence revealing no difference in redilation behavior during bilateral or unilateral photic stimulation.

### Pupil response symmetry (PRS) coefficient

**Figure 7:**
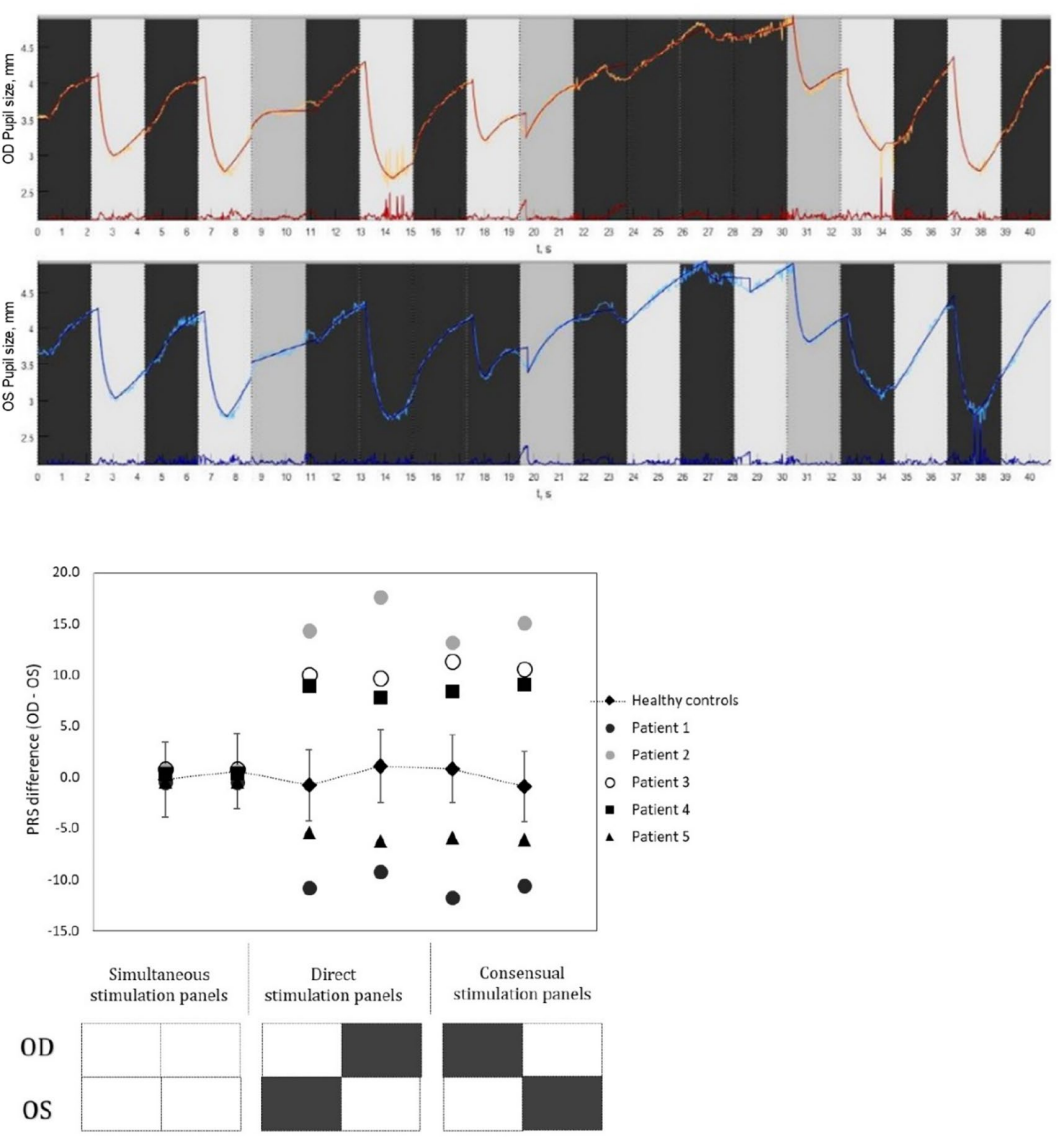
The top panel displays the generated pupil traces from a patient with clinically detected RAPD. Pupillogram showed normal pupil traces during the simultaneous stimulation phase (Sim OU) and unilateral stimulation phase OD (Uni OD), but during the unilateral stimulation phase OD showed no constriction responses from the pupil on light stimulation which was persistent during the Alternating stimulation phase (Alt OD and Alt OS) as well. The bottom panel shows the scatter plot generated using the mean inter-pupillary difference in PRS coefficient values for the healthy subjects (Error bar represents the SD) and the five patients with clinically diagnosed RAPD. The X axis represents the stimulation panels whereas the Y axis displays the inter-pupillary difference in PRS.

The diameter and time-related constriction parameters obtained from all the six light stimulation panels were used to calculate the PRS coefficients for both healthy controls and patients with RAPD. The descriptive summary of those parameters are enumerated in Table 3. The mean (SD) inter-pupillary difference in PRS coefficient values for the healthy subjects (irrespective of their iris colour grades) obtained from the six constriction panels that ranged from − 0.20 to + 1.07 (SD ~  ± 4.0). Figure 7 illustrates distinctly higher inter-pupillary difference in PRS coefficients for the three RAPD patients across all the stimulation panels that are noticeably outside the expected normative limits. The laterality of the afferent defect was evident from the direction of the PRS difference, for example, Patient 1 was clinically diagnosed with grade III RAPD in the left eye whereas patient 2 and 3 were presented with grade III and II RAPD respectively in right eye (Fig. 7).

**Table 3:** Displays the descriptive summary of the constriction parameters obtained for healthy controls and RAPD patients from all the six light stimulation panels.

Mean Constriction parameters		Panel 2	Panel 4	Panel 7	Panel 9	Panel 12	Panel 14
Healthy Controls(n = 29)	d_pup.init_ (mm)	4.6 (0.7)	4.4 (0.3)	4.5 (0.4)	4.6 (0.4)	4.3 (0.4)	4.5 (0.3)
	d_pup.min_ (mm)	2.7 (0.4)	2.6 (0.3)	2.7 (0.3)	2.8 (0.4)	2.4 (0.3)	2.6 (0.3)
	Δd (mm)	1.9 (0.4)	1.8 (0.3)	1.8 (0.3)	1.8 (0.3)	1.9 (0.4)	1.9 (0.4)
	Δt(ms)	255 (30)	260 (30)	251 (35)	250 (50)	240 (40)	245 (35)
	T(ms)	250 (40)	260 (50)	250 (40)	250 (50)	260 (60)	255 (40)
	RMSE	0.04 (0.1)	0.05 (0.1)	0.08 (0.1)	0.10 (0.1)	0.12 (0.1)	0.07 (0.1)
RAPD patients (n = 5)	d_pup.init_ (mm)	4.4 (0.3)	4.4 (0.3)	4.4 (0.3)	4.2 (0.4)	4.2 (0.4)	4.1 (0.4)
	d_pup.min_ (mm)	2.9 (0.3)	2.8 (0.4)	3.5 (0.3)	3.3 (0.4)	3.2 (0.3)	3.1 (0.4)
	Δd (mm)	1.5 (0.3)	1.6 (0.4)	0.9 (0.3)	0.9 (0.4)	1.0 (0.4)	1.0 (0.3)
	Δt (ms)	240 (40)	230 (40)	230 (30)	235 (30)	240 (45)	235 (30)
	T (ms)	240 (35)	245 (35)	220 (30)	225 (35)	235 (35)	230 (35)
	RMSE	0.04 (0.1)	0.04 (0.0)	0.11 (0.1)	0.07 (0.1)	0.06 (0.1)	0.07 (0.1)

## Discussion

The current study described the design and development of a haploscope-based binocular pupillometer. The proposed system incorporated a stimulus presentation paradigm that replicated the SFT. The double exponential fit function aided in the comprehensive parametrization of the PLR and good repeatability of diameter and time-related values were found. The suggested method was found to be feasible and applicable in describing the pupillary dynamics among healthy controls as well as patients diagnosed with RAPD. The evaluation of the effect of iris colour on pupillary dynamics showed that the initial PD (dilation phase) and minimum PD (constriction phase) depended on iris colour whereas no statistically significant differences were found with respect to other parameters. The results showed that the constriction response had a faster course than the dilation and also demonstrated the temporal dynamics of the redilation phase.

In SFT, the pupil reactivity is described with terminologies that relate to the laterality (unilateral/bilateral), pupil size (miotic/mid-dilated/dilated), and reactivity (brisk/sluggish/non-reactive) that is often applied in the absence of any standardized clinical protocol or consistent definition^1–7^. Similar to other automated pupillometry devices, our binocular system, generated the quantifiable outcome measures for PLR under well-controlled lighting conditions including the amount, angulation/direction, and duration of photic stimulation. Hence, this system showed good repeatability of the measurements, resolved the issue of variability resulting from the examiner-dependent errors, and reduced the false-negative responses.

### Pupil dynamics during dilation, constriction, and redilation responses

The current study showed the comparative analysis of time-related pupil parameters during dilation, constriction, and redilation where the dilation phase had higher time constants (Figs. 4, 6) and latency values (Fig. 5) when compared to constriction phases. That means constriction due to light onset happened significantly faster i.e. approximately 5 times (5.3 × times) than the dilation due to light offset. These findings are in par with the literature concerning the description of the physiological trait of pupil constriction as an involuntary reflex mechanism to safeguard retinal cells from phototoxic damage whereas the less well understood neural pathway of dilation in this setting is a passive process when adapting to low levels of illumination^1,2^. The pupil tends to constrict muscularly and briskly until it reaches its minimum size, and remains in the fully constricted state for a while and slowly dilates which is termed as the redilation here. The time constant for this redilation response was found to be approximately two-times (1.9 × times) higher than constriction but lower than dilation, a rapid pupil recovery process after reaching the maximum constriction plateau of sphincter muscle whereas the dilation process is rather a passive visual adaptation process to dark/low levels of illumination. The nature of these temporal characteristics in accordance with the reported pupil physiology acts as a considerable evidence for the rightness of the analysis method we have adopted. The precise quantification of the time courses of pupil response would aid in an improved description of the brisk/sluggish/ill-sustained pupillary reaction in a quantifiable manner unlike the qualitative documentation during the routine clinical practice. The temporal parameters of dilation quantified in this study is probably unique to this experimental set up. The values can be anticipated to be altered if conditions of increased intellectual activity, levels of arousal, wakefulness, and the fight-or-flight response termed as the psychosensory pupil response are applied^1–7^.

### PLR parameters as a function of iris colour

In addition to the level of retinal illumination, accommodative status, and various emotional conditions, iris colour is also reported to have an influence on PD^5,25–28,31,32^. The difference in iris colour among healthy individuals occurs as a result of variable quantities of melanin pigment granules in the superficial stroma of the iris. A previous study quantified PD during a steady light adaptation using an objective infrared-based continuous recording technique and investigated the effect of various factors on pupil size. They reported a dependency of PD on chronologic age whereas it was independent of the gender, refractive error, or iris colour among healthy humans^5^. A previous study that evaluated the effect of iris colour on the Dark-Adapted Pupil Diameter (DAPD), showed no difference in PD between light and dark coloured iris groups and concluded that iris colour does not seem to have a significant role in determining pupil size^28,31^. In contrast, a few other studies found a significant difference in PD between different iris colour grades under various lighting conditions^25,26,32^. These contradictory findings in the literature were our basis for aiming at an evaluation of the quantified PLR parameters between the dark and light iris groups. We found that other than the initial PD during dilation (Table 1) and minimum PD during constriction (Table 2), the rest of the parameters were comparable between the two iris groups. This confirmed that the amplitudes and time-related parameters (constriction and dilation) can be clinically used and relied upon irrespective of the iris colour grades. Since the difference in the diameter-related parameters can possibly be attributed to the variation in the density of melanin pigments/number or sensitivity of autonomic receptors^26^, a significant change in the current findings is not expected, even if we apply a baseline correction for standardization. Bergamin et al.^27^ also demonstrated the dependency of contraction amplitude and velocity on iris colour and suggested that iris colour to be considered as a factor while evaluating pupil movements which was not confirmed in our study.

### Binocular pupillometer system: stimulation and image processing module

Literature has described various binocular pupilometers with unique stimulus projection and image capturing modules to estimate the PLR characteristics by quantifying reliable pupil parameters and evaluating their mutual interactions^14–19^. Meanwhile, certain other studies further explored the possibility of such pupillometer systems (commercially available or lab-based prototypes) to detect and quantify Relative Afferent Pupillary Defects (RAPDs) that showed promising results with a high positive correlation with conventional methods i.e. SFT and Neutral Density Filter^19,20,33,34^.

In contrast, in this study, we adopted a haploscopic principle^22–24^ to design a pupillometer system that can offer a more extensive evaluation of pupil dynamics during bilateral, unilateral, and alternating light and dark stimulation thereby acquiring direct and consensual reflexes concurrently. The captured pupil signals were further processed using a custom-written algorithm to generate PLR curves. Since the changes in pupil size were non-linear (Fig. 3) the PLR traces were fitted with double-exponential growth or decay curves for dilation and constriction respectively, aiding in the quantification of a substantial list of pupil parameters including latency and time constants. Double exponential equations were chosen as it is a promising empirical method to characterize the temporal aspects of the spontaneous fluctuations of biological signals^35^. In the present study, the use of exponential function adds value for the accurate calculation of the PRS coefficients.

Generally, the functional evaluation using electrophysiological methods emphasizes the speed of afferent conduction, so likewise even in pupillometry techniques, the pupil latency and time constants describe the abstract summation of afferent and efferent times of the pupillary pathway until the pupil initiates a response^16,36^. Literature has put forward evidence that pupil latency is delayed in patients with afferent diseases, such as demyelinating disease, optic atrophy, and disorders such as diabetes that affect the efferent autonomic innervation to the iris. Latency, when compared with diameter-related parameters, is reported to be less variable and influenced by the muscular properties of the iris that are known to restrain the drive of the pupil that occurs after the onset of contraction. The exponential fitting procedures aided us in reliable quantification of these temporal aspects of the pupillary dynamics (latencies and time constants) thereby promising objective indices for disease diagnosis and progression assessment^16,35,36^.

### Clinical applicability of the haploscope based binocular pupillometer

We observed that the method demands no expert skill from the examiner because the system is relatively simple to use and the procedure was reported to be convenient by the participants due to its quick and non-invasive nature. That means the features of the prototype and the procedure is well placed for the inclusion of the system in screening assessments and could complement other clinical evaluations to identify individuals at risk who warrant further investigations. However, this prototype requires further considerations and research to enable translation into clinical/community settings. For instance, neurological/neuro-ophthalmic conditions that influence the integration of parasympathetic and sympathetic stimulation and inhibition may affect the dynamics of the PLR. Among various such pupil abnormalities, RAPD is a key clinical sign detected using SFT, which displays an unequal inter-pupillary reaction corresponding to underlying asymmetric damage in the afferent pathways. Here is the relevance of introducing the PRS coefficient considering not only the diameter-related features of the pupil (amplitude) but also the constriction latency (Fig. 5) and time constant (Fig. 4). The descriptive comparison of the diameter and time-related parameters obtained during the constriction panels (Table 3) for the healthy controls and RAPD patients showed markedly smaller pupillary amplitudes (change in PD) among the patients. Though not statistically significant, the Tcon values (Time constants in ms) were numerically faster for the RAPD patients when compared to the controls. This might be linked to the faster but weak initial constriction response followed by a greater redilation that is clinically observed during SFT while examining Grade 1 RAPD patients. In the present study, in addition to visual observation, we have quantified this characteristic behaviour in grade 1 RAPD patients. Still, this would require further inclusion of patients for confirming these findings. A combination of the parameters obtained during constriction responses served as a basis for the calculation of PRS coefficients in which distinctive limits were found between healthy and patients with RAPD. The PRS related assessment could function as a preliminary evaluation towards the establishment of an asymmetry limit to predict the presence of RAPD. But then again in cases of severe grades of RAPD, we speculate there might be no attempt for pupil constriction, rather an instant dilation on light stimulation, then we might have to assume tcon close to zero in order to avoid a PRS coefficient of infinity (∞). Hence, the limits of this coefficient should be further explored in patients with different grades of RAPD to evaluate its clinical applicability. Additional research is also necessary to establish whether the extracted PLR parameters are satisfactory in terms of specificity and sensitivity to be used diagnostically.

Since the pupilometer system we used has haploscopic optics for stimulus presentation, it offers the possibility of evaluating the Pupillary Near Reflex dynamics thereby expanding the applicability to detect Light-near dissociation signs. This could aid in the detection of clinical conditions such as Adie’s tonic Pupil, and Argyll Robinson’s pupil. The customizable protocol offers the opportunity to explore the possibility of chromatic pupillometry and acquire knowledge regarding the Post Illumination Pupillary Response (PIPR) to detect abnormalities in the function of ipRGCs^37–39^. This can aid as a promising diagnostic and therapeutic outcome measurement tool to assess the functional integrity of the inner retina independent of visual photoreceptor input. Further enhancement in the design of this current lab-based prototype can promote it into a portable device and finer modifications in its interface might even open up possibilities for a tele-ophthalmic evaluation of clinically relevant pupil signs. Even though, the current study has attempted to take care of various factors influencing PLR measurements, a variety of emotional and cognitive elements should be considered as covariates. Moreover, there was no availability of other pupillometer systems for a preliminary comparative analysis.

## Conclusions

The current study proposed an automated haploscope-based binocular pupillometer system. This provides insights into analysis strategies that could be successfully applied to optimize the quantification of PLR parameters. The clinical relevance of this protocol can be further explored in detecting the pupil abnormalities in various neuro-ophthalmic diseases, and in utilizing the characteristics of pupil redilation in disease detection. Further steps can focus on optimizing the PRS coefficient for quantifying afferent pupillary defects.

## Supplementary Information

Supplementary Information 1.

Supplementary Information 2.
