# Supplementary material for: A haploscope based binocular pupillometer system to quantify the dynamics of direct and consensual Pupillary Light Reflex
Source: Sci Rep. 2021 Oct 26;11:21090. doi: 10.1038/s41598-021-00434-z (PMC8548319; doi:10.1038/s41598-021-00434-z)
Supplement: Supplementary file 1 — Supplementary Information 1. [file 41598_2021_434_MOESM1_ESM.docx]

**Appendix 1:**

The double exponential equations used to analyze the time course of the pupil trajectory and the quantify PLR parameters:

d_pup.init (c)_ - The initial pupil diameter in a panel, d_pup.max (c)_ - The maximum pupil diameter in a dilation panel, d_pup.min (c)_ - The minimum pupil diameter in a constriction panel, d_pup.end (c)_ - The pupil diameter at the end of redilation period in a constriction panel, Δd - difference between d_pup.max_  or d_pup.min_ and the d_pup.init._, Δt_dil_ - The latency for the initiation of dilation after light stimulation is of, Δt_con_ - The time delay/latency for the initiation of constriction after light stimulation is on, Δt_rec_ - The time delay/latency for the initiation of pupil redilation after the maximum constriction, T_dil,_ T_con_, T_rec_ - the time constants (tau)

RMSE – Root Mean Square Error

PRS - Pupil Response Symmetry

$d_{pup.max} = d_{pup.init}+ {\Delta d}_{\mathrm{dil}} \left( 1-\exp\left[ -\frac{t-{\Delta t}_{\mathrm{dil}}}{T_{\mathrm{dil}}} \right] \right)$ (eq. 1)

$d_{pup.min} = d_{pup.max}- {\Delta d}_{\mathrm{con}} \left( 1-\exp\left[ -\frac{t-{\Delta t}_{\mathrm{con}}}{T_{\mathrm{con}}} \right] \right)$ (eq. 2)

$d_{pup.end} = d_{pup.min}+ {\Delta d}_{\mathrm{red}} \left( 1-\exp\left[ -\frac{t-{\Delta t}_{\mathrm{red}}}{T_{\mathrm{red}}} \right] \right)$ (eq. 3)

$t_{\mathrm{con}} = {\Delta t}_{\mathrm{red}}- {\Delta t}_{\mathrm{con}}$ (eq. 4)

RMSE =$\sqrt{\frac{\sum_{i=1}^{N} {(predicted\left( i \right)-actual\left( i \right))}^{2}}{N}}$ (eq. 5)

PRS = $\frac{\frac{T_{\mathrm{con}}}{t_{\mathrm{con}}}\times100}{{\Delta d}_{\mathrm{con}}}$ (eq. 6)
