# Supplementary material for: A haploscope based binocular pupillometer system to quantify the dynamics of direct and consensual Pupillary Light Reflex
Source: Sci Rep. 2021 Oct 26;11:21090. doi: 10.1038/s41598-021-00434-z (PMC8548319; doi:10.1038/s41598-021-00434-z)
Supplement: Supplementary file 2 — Supplementary Information 2. [file 41598_2021_434_MOESM2_ESM.docx]

**Supplementary Figure 1:** Bar graphs displaying the comparable Time constant values (top panel) and latencies (bottom panel) between the six constriction panels. The error bars represent the Standard Deviation (SD).

**
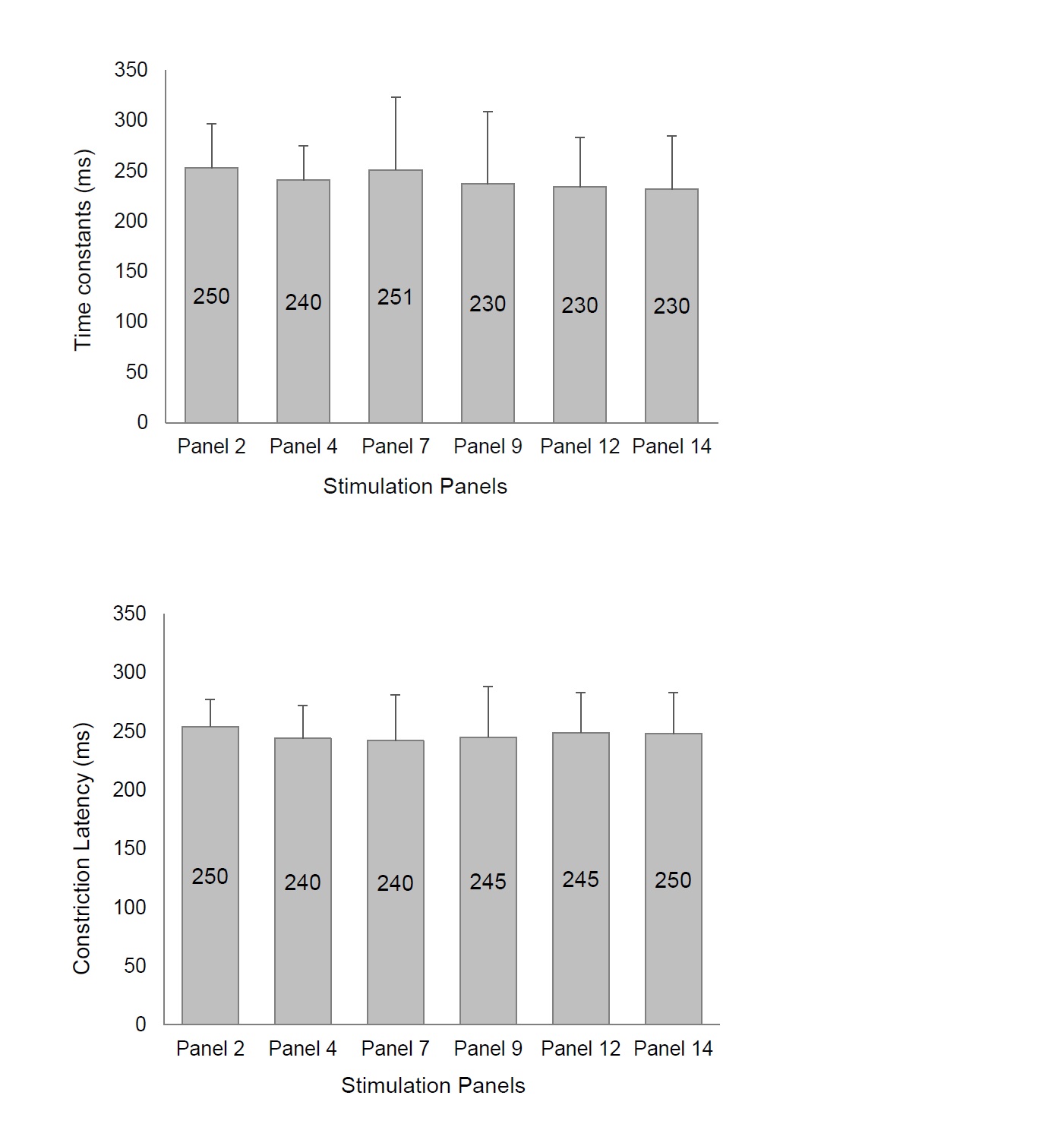
**
